# The need for preventive and curative services for malaria when the military is deployed in endemic overseas territories: a case study and lessons learned

**DOI:** 10.1186/s40779-017-0128-3

**Published:** 2017-06-06

**Authors:** Sumadhya Deepika Fernando, Rahuman Booso, Priyani Dharmawardena, Arunagirinathan Harintheran, Kugapiriyan Raviraj, Chaturaka Rodrigo, Manjula Danansuriya, Rajitha Wickremasinghe

**Affiliations:** 1 Department of Parasitology, Faculty of Medicine, University of Colombo, Colombo, Sri Lanka; 2 Directorate of Health Services, Sri Lanka Air Force, Colombo, Sri Lanka; 3 Anti-Malaria Campaign, Ministry of Health, 555/5 Public Health Complex, Narahenpita, Colombo, 5 Sri Lanka; 4 Department of Pathology, School of Medical Sciences, University of New South Wales, Sydney, NSW 2052 Australia; 5 Department of Public Health, Faculty of Medicine, University of Kelaniya, Kelaniya, Sri Lanka

**Keywords:** Imported malaria, Security forces, Sri Lanka, Central African Republic, Chemoprophylaxis

## Abstract

**Background:**

Sri Lanka has been free from indigenous malaria since November 2012 and received the WHO certificate for malaria-free status in September 2016. Due to increased global travel, imported malaria cases continue to be reported in the country. Military personnel returning home from international peace-keeping missions in malaria endemic countries represent a key risk group in terms of imported malaria. The present study intended to characterize the potential causes of a malaria outbreak among the Sri Lankan security forces personnel deployed in the Central African Republic (CAR).

**Methods:**

Data were collected from a cross-sectional survey distributed among Sri Lankan Air Force personnel who had returned from United Nations peace-keeping missions in the CAR region. A pre-tested questionnaire was used for the data collection, and focus group discussions were also conducted.

**Results:**

One hundred twenty male Air Force personnel were interviewed (out of a group of 122 officers and airmen). All participants were deployed in the CAR for 14 months and were aware of the existence of chemoprophylaxis against malaria. The majority of the subjects (92.5%, 111/120) also knew that prophylaxis should be started prior to departure. However, the regular use of chemoprophylaxis was reported by only 61.7% (74/120) of the sample. Overall, 30.8% of the participants (37/120) had 44 symptomatic episodes of malaria during deployment, and one person succumbed to severe malaria. All cases were associated with noncompliance with chemoprophylaxis.

**Conclusion:**

Better coordination with overseas healthcare services and the establishment of directly observed chemoprophylaxis may help to avoid similar outbreaks in the future.

## Background

With no indigenous malaria cases having been reported since November 2012, the World Health Organization declared Sri Lanka a malaria-free country in September 2016, a milestone for a tropical country with a population of approximately 22 million [1]. The biggest challenge faced by the country today is the prevention of the re-introduction of malaria due to imported malaria cases.

During the years prior to the cessation of transmission, i.e., 2008–2012, approximately 75% of indigenous malaria cases reported in the country were from the military. From 2008 to 2012, 223 imported malaria cases were reported in Sri Lanka, and 16 of these were from military personnel returning from malaria-endemic countries. Sri Lanka has been a frequent contributor to United Nations (UN) Peacekeeping forces, deploying its troops to destinations such as Haiti, Sudan, the Central African Republic and Liberia. All of these countries are endemic for malaria, and their local health infrastructure is severely affected due to ongoing conflicts. The importation of malaria to currently malaria-free Sri Lanka by security forces personnel returning home is a major concern, and measures have been expanded to face this challenge [2]. Chemoprophylaxis is usually issued by the Ministry of Health of Sri Lanka or the UN missions overseas, but during long-term deployments, as reported in this manuscript, difficulties have been encountered in the acquisition of appropriate chemoprophylaxis in war-torn foreign destinations. The present study intended to characterize the potential causes of a malaria outbreak among the Sri Lankan security forces personnel deployed in the Central African Republic and to recommend preventive measures to avoid a similar outbreak in the future.

## Methods

A cross-sectional survey was distributed to Air Force personnel who had returned after a United Nations Peace-keeping mission in the CAR region from the 9th of September 2014 to the 10th of November 2015 (14 months). A pre-tested interviewer administered the questionnaire that was used for data collection. Interviews were conducted by a research team who had not been serving in military establishments. This quantitative assessment was accompanied by focus group discussions that were conducted among individuals who had contracted malaria during deployment to enable in-depth discussions of the participants’ perceptions relative to the cause of malaria outbreaks.

The globally recommended chemoprophylactic drugs for visitors to the CAR are mefloquine, doxycycline and atovaquone-proguanil [3]. Chemoprophylaxis was considered a) regular if the drugs had been taken for the whole duration of the stay abroad at the recommended doses and intervals, b) irregular if chemoprophylaxis had been started and taken at a lesser frequency than recommended, but not completely stopped, or c) not performed if chemoprophylaxis had been started and subsequently stopped or had never been started.

### Statistical analyses

The data were analyzed using the SPSS (Version 20, IBM, USA) statistical package. Descriptive statistics were used to describe the distribution of the important variables identified. Statistical associations for single or multiple attacks of malaria were assessed via the chi-square test.

## Results

Out of the 122 airmen and officers that had left Sri Lanka for the UN Mission, 120 (98.4%) were interviewed. One person was not present on the day of the interview and the other had died while overseas due to severe malaria. All participants were males (36.5 ± 5.2 years) from the Sri Lanka Air Force and had been deployed in the CAR for 14 months. They did not have any other history of overseas travel within the year preceding the deployment. A total of 15 officers (12.5%, 15/120) were in the group. All of the interviewed men were aware of the existence of chemoprophylaxis against malaria. Most were also aware that prophylaxis had to be started prior to departure (92.5%, 111/120), needed to be taken regularly while in endemic areas (98.3%, 118/120), and should be continued for a defined period of time after return (97.5%, 117/120). The drugs had been provided to all airmen by the Air Force medical corps. Eighty airmen (66.7%, 80/120) received the first dose prior to departure, but the remainder received it after arrival in the CAR. Despite knowing that chemoprophylaxis had to be taken regularly while in high-risk areas, only 61.7% (74/120) of the subjects followed the correct practice. Others reported irregular use, but none had totally abandoned chemoprophylaxis. When asked about the instructions on how they consumed the drugs, only 78 (65.0%, 78/120) individuals were able to correctly repeat the advice.

The troops had only a limited supply of mefloquine (the drug of choice) when they left Sri Lanka. This was not sufficient to maintain prophylaxis for everyone throughout the deployment. Therefore, 82 individuals (68.3%, 82/120) reported a change of medication to doxycycline following arrival (after 2 months). Another person had preferred doxycycline because he could not tolerate mefloquine. Side effects, mainly gastrointestinal complaints followed by joint pains attributable to chemoprophylaxis, were reported by 55 airmen (45.8%, 55/120). No one suffered from the neuropsychiatric side effects of mefloquine despite taking the drug for over 12 months.

A further analysis was carried out only in the individuals who contracted malaria. Overall, 37 participants had 44 symptomatic episodes of malaria during deployment, with 31 participants who had one attack and six who had more than one attack of malaria (one individual had three attacks and five individuals had two attacks) (Table 1). Two peaks in the malaria incidence occurred over the period of stay, one in November 2014 (one month after deployment) and the other in June–July 2015 (9–10 months after deployment). Information regarding diagnosis and species were taken from the records maintained by the Air Force (Table 1). Of the 37 subjects who contracted the disease, twelve individuals took mefloquine throughout their stay, and the others had changed to doxycycline after 2 months of arrival in the CAR. However, all individuals who contracted malaria reported non-compliance with chemoprophylaxis (regardless of whether it was mefloquine or doxycycline). The main reason for this was that they were deployed in the field most of the time and forgot to take the drugs. Other characteristics and treatment-related issues regarding the malaria attacks are summarized in Table 1.

**Table 1:** Descriptive statistics of the malaria attacks during deployment (*n* = 44)

Characteristic	Number	Percentage (%)
Number of attacks		
One	31	83.8
Two	5	13.5
Three	1	2.7
Species		
*Plasmodium falciparum*	10	22.7
*Plasmodium vivax*	6	13.6
Not recorded/do not know	28	63.7
Diagnosed by		
Rapid diagnostic testing	42	95.5
Microscopy	2	4.5
Number of days from the onset of symptoms to the diagnosis of malaria		
1	15	34.1
2	2	4.6
3	7	15.9
4	2	4.6
5–7	1	2.2
Not recorded	17	38.6
The number of days from diagnosis to the start of treatment		
1	21	47.7
2	1	2.3
3	5	11.4
4	2	4.5
5–7	3	6.8
Not recorded	12	27.3

Forty-four includes all attacks of malaria that occurred during the period of deployment

Apart from chemoprophylaxis, adherence to other prevention strategies was also poor. Most participants had not used or irregularly used mosquito nets (52.5%, 63/120), repellents (90.8%, 109/120), and mosquito coils (98.3%, 118/120) and had not worn long-sleeved clothing (89.2%, 107/120) due to practical difficulties.

We also assessed the statistical associations for the contraction of at least one attack of malaria or multiple attacks of malaria. Regarding the demographic parameters, the entire sample consisted of males who belonged to the same ethnicity (Sinhalese) and had completed secondary education. Of the other variable parameters, the rank in the air force, having stayed in Bangui, having stayed in Bria, having a transit in Bangui, having had the first dose of chemoprophylaxis after departure or switching the prophylactic drug did not show any significant associations with having a malaria attack. However, not adhering to regular chemoprophylaxis (*P* < 0.001) and experiencing adverse events with chemoprophylaxis (*P* = 0.05) were both associated with contracting malaria. When comparing a single attack vs. multiple attacks of malaria, none of the above factors were predictive of the latter outcome.

In the absence of recommended drugs such as artemisinin-based combination therapy (ACT) [4], when diagnosed with malaria, patients were managed according to the locally developed guidelines. For uncomplicated vivax malaria, doxycycline and quinine were given orally for 7 days followed by primaquine for 14 days. Severe malaria was treated with intravenous quinine. Three cases of severe malaria were observed within this group, and one subject died.

Regarding the death, the victim was a 38-year-old airman who did not take chemoprophylaxis due to gastrointestinal side effects. He presented with nausea and vomiting for two days and was diagnosed with *falciparum* malaria based on the rapid diagnostic test. Treatment was commenced for uncomplicated falciparum malaria with oral quinine and doxycycline, but his condition worsened within the next 24 h. Intravenous quinine was started immediately (without a loading dose). No clinical improvement was observed in the next 24 h, and he was transferred to a higher-tier hospital where he developed atrial fibrillation, which was probably influenced by the high quinine dose. The attending doctors recommended an urgent blood transfusion (no record of the hemoglobin level at this point), but no facilities were available for transfusions in that hospital. The patient was being airlifted to a higher-tier hospital in Uganda when he suffered a cardiac arrest and passed away on the 4th day of the illness.

During the focus group discussions, some important themes emerged. Knowledge regarding the need for chemoprophylaxis to prevent malaria was adequate amongst the troops. However, the circumstances of the working environment were such that they could not put the knowledge into practice. The recommended chemoprophylaxis (mefloquine) was not available in adequate quantities and artemether-lumefantrine, which is the recommended drug for the treatment of malaria, was unavailable at the destination. Of the recommended drugs, only quinine was available for the treatment of severe falciparum malaria [3]. Only primary care facilities were available on-site near the camps. The primary care center was well-staffed, with 2 doctors, 8 nurses and a pharmacist. Patients with severe illness were transferred to a higher tier hospital, but the recommended treatment recourses were limited in these hospitals as well. Prior to departure, the troops were advised regarding the prevention of, and the prophylaxis for, malaria and HIV/AIDS. All were aware of other malaria preventive strategies such as the use of repellents, wearing long-sleeved clothes, etc., but many admitted that most of these measures were not practiced even after an outbreak due to the nature of the occupation. They also had a false sense of security, since the camps were sprayed with an insecticide (permethrin) once every three months. In addition, during the first 4 months of the deployment, the troops were housed within temporary shelters and they worked outdoors, constructing their permanent shelters while being exposed to mosquito bites. The number of cases had declined by December 2014 when the permanent shelters were completed.

Upon the return of this group to Sri Lanka in November 2015, the malaria screening program conducted by the anti-malaria campaign at the Bandaranaike International Airport detected two further falciparum malaria cases by rapid diagnostic testing, which were later confirmed as being positive for malaria by microscopy. The recovery of both individuals was uneventful. Thereafter, all returnees were screened 6 weeks after arrival to ensure diagnoses of asymptomatic malaria infections. All tested negative for malaria by rapid diagnostic (antigen) testing and microscopy. At the follow-up of these individuals 4 months later, another malaria-positive person was diagnosed by microscopy and the rapid diagnostic test despite testing negative in previous screenings. This was a relapse infection due to *P.ovale.*

## Discussion

Since the last indigenous malaria case was reported in 2012, the main population at risk for developing malaria in Sri Lanka has been the security forces personnel who serve in the conflict-affected districts of Sri Lanka, which also happen to be the districts with high incidences of malaria. Sri Lanka endured a separatist war for nearly 30 years from 1981 to 2009 and after the cessation of hostilities in 2009, the soldiers who had been involved in combat focused their efforts on reconstruction and rehabilitation. Additionally, an increased number of troops were deployed on UN Peace-keeping missions overseas. The partnership between the anti-malaria campaign (AMC) and the Ministry of Defense ensures that all returnees are screened for malaria at the ports of entry to Sri Lanka. The medical corps of all three armed forces (Sri Lanka Army, Navy and Air Force) readily cooperates with the AMC, and its officials attend the monthly review meetings of the AMC. The AMC also provides training to the laboratory staff of the medical corps on malaria diagnosis, treatment and follow-up and provides guidance on policies and strategies for malaria control in the security forces.

Due to the chemoprophylaxis regimen that was followed by security forces personnel under the guidance of the AMC, the number of malaria cases reported among security forces personnel returning to Sri Lanka after deployment abroad was less than 6% of the total reported malaria cases between 2013 and 2016 (12 cases/219 imported cases). The continuation of the sustained, intensive surveillance mechanism that is currently in place is essential for the prevention of the re-introduction of malaria to Sri Lanka. The armed forces and the police inform the AMC of their personnel returning to the country from foreign missions well in advance of their expected date of return and on every such occasion, the AMC will screen the returnees at the airport and follow-up with them for several months with the cooperation of the respective medical corps. The re-introduction of malaria can have extensive consequences in Sri Lanka because the population has not been exposed to the disease for a long time. Even the doctors are less experienced in diagnosing malaria because many have not seen a patient with malaria [5]. Despite the interruption in the transmission of the parasite, the mosquito vector is still abundant in Sri Lanka [6].

This study summarizes a quantitative and qualitative assessment of the largest reported malaria outbreak among Sri Lankan peacekeeping forces deployed overseas in the last 5 years. The troops were deployed to the Central African Republic, a country that has been subject to sectarian violence that started in the township of Bangui in December 2013 [7]. An estimated 72% of the country’s health facilities are currently non-functional [7]. One hundred and fifty-eight million people in the ten countries of this sub region are at risk for malaria, with 145 million at a high risk. Cases are almost exclusively due to *Plasmodium falciparum* malaria. Malaria is the leading cause of death in children under 5 years of age and accounts for up to 60% of outpatient consultations in the Republic [7]. The Sri Lankan forces had been deployed in the townships of Bria and Bangui, which are some of the worst affected regions by violence. The Centers for Disease Control and Prevention, USA consider the whole of the CAR to be at a high risk for malaria and recommends either doxycycline, mefloquine or atovaquone-proguanil as chemoprophylaxis [4]. However, there are reports of doxycycline failure in the CAR [8].

When analyzing the outbreak of malaria in Sri Lankan forces in the CAR, several important observations were noted. The troops were educated on the health risks of malaria and the preventive measures prior to departure by the AMC, who routinely provides such advice and guidance to troops [2]. As a result, almost all personnel were aware of the correct method of taking chemoprophylaxis and of the other malaria preventive strategies. Chemoprophylaxis was issued free of charge by the AMC and was initiated prior to departure by the medical corps of the Air Force in two-thirds of the group and the remainder received the first dose of chemoprophylaxis after arrival in the CAR. They were given doxycycline, which is a recommended drug for malaria chemoprophylaxis in the CAR, when sufficient stocks of mefloquine were unavailable. Upon return to the country, all personnel were screened for malaria at the airport, which led to the detection of 2 further cases. The importance of further follow-up with these individuals on a regular basis is highlighted by the detection of a *P.ovale* case 4 months after their return.

However, lapses were observed in the conversion of knowledge to practice, which probably led to the occurrence of malaria in 31% of the airmen who were deployed and also resulted in 1 death. Despite their awareness of the risk, many chose to ignore the advice due to unfavorable climatic conditions for the appropriate attire and due to the stresses of active military service in a conflict zone. This situation was further complicated by logistic issues such as exposure to mosquito bites in temporary shelters. For chemoprophylaxis, even though sufficient stocks of doxycycline were made available once the mefloquine ran out, the compliance was probably poor given the daily dosing requirement. This could be prevented in the future by administering the chemoprophylactic drugs under direct observation. Delays between the onset of symptoms and medical care were observed because the troops that were in the field had to return to base camp where the medical staff was located. More importantly, the lack of advanced medical facilities to manage severe cases of malaria is a cause of serious concern. In this case study, several failures were observed. First, falciparum malaria patients were not treated with ACTs as recommended by the WHO due to the unavailability of the drug. Second, basic emergency medical support for the life-threatening complications of malaria such as renal replacement therapy and blood transfusions were difficult to arrange. These issues need to be urgently corrected to prevent further morbidity and mortality among military personnel deployed in UN missions in the malaria-endemic CAR.

The issue of administering malaria prophylaxis to long-term visitors to countries that do not routinely advocate the use of prophylaxis due to high transmission rates warrants investigation by accompanying medical officers. Although the administration of malaria chemoprophylaxis to short-term visitors to these countries is justifiable, other preventive measures should also be taken into consideration, particularly among long-term visitors who stay for more than 6 months. For example, proven preventive measures such as the administration of chemoprophylactic drugs with a directly observed treatment strategy or the use of insecticide-treated nets (whenever possible) could have been more useful [9].

The economic and social costs of the re-introduction of malaria to Sri Lanka can be huge and can be as devastating as any man-made conflict or civil war. During an epidemic in 1934–1935, an estimated number of 80,000 Sri Lankans died of malaria within 7 months [10]. With decreased external financing and the political interest in sustaining malaria surveillance within the country, continued surveillance at the ports of entry is critical to the prevention of re-introduction. Therefore, while contributing to our international obligations, the implementation of measures to safeguard the country and its servicemen from malaria contracted overseas is of utmost importance.

## Conclusions

The malaria outbreak described in this paper among Sri Lankan Air Force personnel was the result of exposing troops with no immunity to malaria to an area of intense malaria transmission without proper healthcare facilities to diagnose and treat malaria. The findings of the present study highlight the importance of ensuring the availability of adequate stocks of malaria diagnostics, anti-malarial drugs and trained staff onsite to diagnose and treat malaria during overseas UN missions. Ensuring adherence to chemoprophylaxis, possibly by directly observed treatment and surveillance maintenance by trained staff, is essential to the prevention of such outbreaks in the future.

## Abbreviations

ACT: Artemisinin combination therapy
AMC: Anti-malaria campaign
CAR: Central African Republic
UN: United Nations
